# Personalized Approach to Chronic Antibiotic-Refractory Pouchitis: A Case Report and Review of the Literature

**DOI:** 10.7759/cureus.53398

**Published:** 2024-02-01

**Authors:** Mili Shah, Aarshdeep Masson, Hamsika Moparty, Dhir Gala, Vikash Kumar

**Affiliations:** 1 Internal Medicine, American University of the Caribbean School of Medicine, Cupecoy, SXM; 2 Internal Medicine, The Brooklyn Hospital Center, Brooklyn, USA

**Keywords:** immunomodulatory therapy, probiotics, mesalamine, ulcerative colitis, carp (chronic antibiotic refractory pouchitis), ileoanal anastomosis, antibiotic refractory, chronic pouchitis

## Abstract

Patients who undergo restorative proctocolectomy and ileoanal anastomosis can develop pouchitis as a common chronic complication. A rare subset of patients fails to respond to multiple antibiotic therapies and develop chronic antibiotic-refractory pouchitis (CARP). We present a case of a 45-year-old male with pouchitis refractory to chronic antibiotic therapy and histology demonstrating chronic inflammatory changes. Management involved mesalamine and probiotics, resulting in a positive clinical response and symptom absence on follow-up. This case highlights the intricacies of treating chronic pouchitis post ileoanal anastomosis, showcasing the efficacy of a personalized approach using mesalamine and probiotics. CARP is emerging as an entity associated with poor quality of life and increased healthcare costs. CARP fails to respond to multiple courses of antibiotic therapy. Therefore, the management of CARP is difficult and limited. Current literature on the management of CARP is scarce and mainly involves immunomodulatory therapy and probiotics. It is essential to keep this differential diagnosis in mind in patients with recurrent pouchitis episodes and start them on immunomodulator treatment and probiotics rather than repeated courses of antibiotics.

## Introduction

Chronic antibiotic-refractory pouchitis (CARP) is a type of inflammatory condition affecting the ileal pouch in patients who have undergone ileal pouch-anal anastomosis (IPAA) procedures for the treatment of ulcerative colitis. The ileal pouch is a surgically implanted artificial pouch that functions to replace the colon. The pouch can be inflamed leading to CARP [1]. Patients with CARP frequently experience symptoms like diarrhea, rectal bleeding, abdominal pain, and a lower quality of life. CARP is challenging to manage and treat because the pouch continues to be inflamed despite antibiotic therapy [2,3]. Because the underlying cause of CARP is not well understood, it is difficult for healthcare professionals to effectively manage the condition.

A thorough analysis of a patient's medical history, symptoms, and examination findings is necessary for the diagnosis of CARP. Antibiotics, immunosuppressants, and biological agents may all be used as part of a CARP treatment plan, which must be customized for each patient based on their unique requirements and medical background [3]. A comprehensive understanding of CARP is crucial for healthcare providers to effectively manage this debilitating condition and improve the quality of life for the patients. We present a case of a 45-year-old male with pouchitis refractory to chronic antibiotic therapy and histology demonstrating chronic inflammatory changes. This case demonstrates the complexities of treating chronic pouchitis after ileoanal anastomosis and the efficacy of a tailored strategy with mesalamine and probiotics.

This case report was previously presented as a meeting abstract at the 2022 American College of Gastroenterology Conference on 24th October 2022.

## Case presentation

A 45-year-old male with a history of ulcerative colitis and previous ileoanal anastomosis presented to the gastroenterology clinic for routine follow-up assessment. At the time of presentation, vital signs were stable and within normal limits, and the physical examination was unremarkable. His ileoanal anastomosis surgery had a complicated postoperative course with recurrent episodes of pouchitis for several years despite several courses of antibiotic therapy. He reported at least three episodes of pouchitis in a year.

In the course of ongoing surveillance, the patient underwent multiple colonoscopies, revealing evidence of mild chronic inflammation within the pouch. Further investigation using magnetic resonance imaging (MRI) of the perineum uncovered small intersphincteric fistulous tracts extending from the anal gland, requiring surgical repair. Notably, inflammatory markers such as C-reactive protein (CRP) and calprotectin levels were within the normal range. Additionally, tests for immunoglobulin G4 (IgG4) and antineutrophil cytoplasmic antibodies returned negative results.

**Figure 1:**
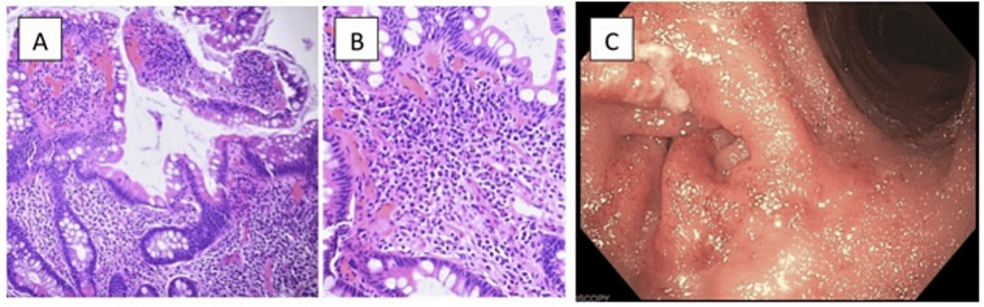
(A & B) Histopathology showing edema and acute and chronic inflammation in the ileal villi. (C) Colonoscopy image demonstrating gross inflammation of the colonic mucosa.

Histopathological examination of the most recent biopsies obtained from the pouch demonstrated moderate chronic inflammation (Figure 1). Magnetic resonance cholangiopancreatography (MRCP) ruled out primary sclerosing cholangitis as a complicating factor.

The patient was managed with a therapeutic regimen involving mesalamine and probiotics. Mesalamine, a known anti-inflammatory agent, and probiotics, aimed at restoring the balance of gut microbiota, were chosen as the treatment strategy to address the observed chronic inflammation and mitigate recurrent pouchitis episodes.

Follow-up assessments revealed a positive clinical response with a reduction in the inflammation within the pouch. The patient reported no further pain or any other symptoms. This case underscores the complexity of managing chronic pouchitis post ileoanal anastomosis in the setting of ulcerative colitis. The tailored therapeutic approach involving mesalamine and probiotics proved effective in achieving clinical remission for this patient.

## Discussion

Individuals with ulcerative colitis may undergo a restorative proctocolectomy and ileoanal anastomosis with a chronic complication of that procedure being pouchitis. Pouchitis is the inflammation of the surgically created pouch, which leads to frequent, watery stools that can be accompanied by other symptoms such as incontinence and even abdominal cramps [4]. Normally, a course of antibiotics can be prescribed to treat this condition; however, CARP is a more severe type of pouchitis that requires antibiotic therapy for more than four weeks and is a result of at least four episodes in a year [5]. This condition is thought to arise due to many factors playing a role, which include but are not limited to bacterial overgrowth, genetics, autoimmune, dysbiosis, and even nutritional deficiencies, which lead to inflammation of the created pouch [4]. Out of these risk factors for CARP, dysbiosis and autoimmune response leading to inflammation are thought to be the main causes leading to this condition [5,6]. Dysbiosis refers to the changes in the microbiome of the gut from a mutualistic relationship with the host to the microbiome adapting a more “pathobiont” relationship leading to other issues such as chronic inflammation [6]. Lastly, the prevalence of chronic pouchitis has been associated with a gender bias as it is more prevalent in males compared to females but the reasons for this are unknown [7].

Furthermore, in terms of the prevalence of CARP, studies have shown this to vary depending on the criteria used for diagnosis [4]. Although an uncommon finding of IPAA, there are many other conditions that can mimic the same symptoms, such as pouch fistulas, strictures, and even irritable pouch syndrome; therefore, it is essential to rule these out before the diagnosis of CARP is made [8]. These findings are not always seen with all individuals undergoing IPAA as CARP is primarily immune-mediated and therefore mainly individuals undergoing the procedure for ulcerative colitis are the ones at the highest risk of developing this condition [9].

When it comes to diagnosing CARP, the modality that is best suited for this is the use of the Pouchitis Disease Activity Index (PDAI), which is a multifactorial diagnostic tool that takes into account clinical, endoscopic, and histological markers to diagnose pouchitis [4]. A score on the PDAI of ≥7 is indicative of pouchitis but is a non-specific determinant if the pouchitis is characterized to be CARP or just acute, in which a course of antibiotics would resolve the issue. Despite being the best method to diagnose the various types of pouchitis, PDAI is not frequently used in a clinical setting due to the scores not being validated and leaving much of the decision up to the clinician in regards to the histopathology and endoscopic findings [4]. Furthermore, additional testing should be performed if it is suspected to be refractory pouchitis such as a pouch biopsy and stool sampling as these findings would not be seen using any of the testing available [4].

Additionally, since CARP lasts for a period of more than four weeks and is quite resistant to antibiotics, the inflammation is severe, leading to an increase in inflammatory cells such as lymphocytes, plasma cells, and eosinophils [10]. All of these cells are characteristic findings of prolonged inflammation, which would occur in patients who have CARP. However, individuals who undergo IPAA due to ulcerative colitis are shown to have an increased susceptibility to an inflammatory response in their pouch compared to individuals who undergo resection due to other conditions such as familial adenomatous polyposis [11,12]. However, if this severe inflammation persists, it has been noted that the mucosa of the pouch will completely alter from its normal appearance. There will be no signs of normal mucosa, but rather it will appear atrophied and resemble the appearance of normal colonic mucosa compared to the mucosa of the small intestine [10]. An additional histological finding that has been observed in patients with an autoimmune disorder along with CARP is the presence of IgG4-positive plasma cells within the mucosa of the pouch [13]. This finding has been supported in other studies that also state IgG4 plasma cells were more frequently seen in patients with CARP when compared to individuals with pouchitis that was not classified as such [5].

In terms of treatment for CARP, it has been shown to respond to many different treatment methods, which vary across studies. Some of the main treatment options include antibiotics, immunomodulators, anti-tumor necrosis factor (anti-TNF), and even probiotics. Although requiring prolonged use of antibiotics, CARP has shown to be responsive in some studies to ciprofloxacin in combination with metronidazole [1,14]. In addition to antibiotics, an immunosuppressive approach has also proven successful in treating the inflammation associated with CARP. The immunomodulator that has shown improvement in patients is azathioprine, with some studies indicating its functionality while others being unsure [15]. This method of treatment should prove successful due to the nature of CARP being associated with an autoimmune disorder; however, a definitive answer is still sought after. When compared to immunomodulators, biologics such as anti-TNF have proven to be very effective with some studies showing remission of CARP with the administration of infliximab in 43% of patients [16]. Lastly, there is the use of probiotics, which can be useful in adjusting the pouch biota to reduce inflammation. Probiotics have been shown to have many effects, which include immunomodulation, including an anti-inflammatory role along with increasing the mucosal barrier, which can be beneficial in preventing further inflammation [6].

Since CARP has been proven to be treated using an empirical approach, the prognosis and outcome vary between patients. It is primarily an immune-mediated disorder, therefore its treatment is very similar to that of inflammatory bowel disease in which suppression of the inflammation is the main target of treatment [15,17]. Depending on the patient, the outcome can be very simple and a dose of antibiotics such as ciprofloxacin may be able to resolve the issue [4]. However, on the other hand, patients may need to undergo treatment using anti-TNF, which can result in the remission of CARP after a period of use in some patients [16]. This ambiguity in treatment seems to suggest that physicians need to treat this condition on a case-by-case basis and work with the patient closely to come to a solution.

## Conclusions

In conclusion, our case report sheds light on the challenging clinical scenario of CARP in a 45-year-old male with a history of ulcerative colitis. Despite the intricacies of managing CARP, our patient exhibited a positive clinical response to a tailored therapeutic approach involving mesalamine and probiotics. This emphasizes the importance of individualized treatment strategies in addressing the chronic inflammation seen after ileoanal anastomosis. Furthermore, our discussion emphasizes the complexity of diagnosing CARP, with considerations ranging from clinical symptoms to histological findings. The multifaceted nature of CARP, including its links to dysbiosis, autoimmune responses, and gender bias, necessitates a nuanced understanding for effective management. Treatment options, ranging from antibiotics to immunomodulators and biologics, further underscore the need for a personalized approach to navigating this immune-mediated disorder. As the prognosis of CARP remains variable and often empirical, this case report contributes to the growing body of evidence, urging clinicians to adopt a case-by-case perspective, collaborate closely with patients, and explore tailored therapeutic interventions to optimize outcomes in the management of CARP.
